# Implant Materials for Anterior Column Reconstruction of Cervical Spine Tumor

**DOI:** 10.1111/os.13702

**Published:** 2023-03-23

**Authors:** Jiasheng Chen, Shuheng Zhai, Hua Zhou, Panpan Hu, Xiaoguang Liu, Zhongjun Liu, Xiao Liu, Yan Li, Zihe Li, Feng Wei

**Affiliations:** ^1^ Department of Orthopaedics Peking University Third Hospital Beijing China; ^2^ Ministry of Education Engineering Research Center of Bone and Joint Precision Medicine Beijing China; ^3^ Beijing Key Laboratory of Spinal Disease Research Beijing China

**Keywords:** 3D‐printed, Allogenic bone, Autogenous bone, Bioactive ceramics, Bone cement, Cervical spine reconstruction, Cervical spine tumor, Implant materials, PEEK, TMC

## Abstract

The spine is the most common site of bone metastases. Many cancer patients will ultimately develop spinal metastatic disease with symptomatic epidural spinal cord compression. At present, the main treatment for cervical spine tumors is surgical resection combined with postoperative radiotherapy. Implant materials for cervical spine anterior column reconstruction need to meet amounts of different properties, such as biocompatibility, bioactivity and the ability to maintain long‐term mechanical strength. The selection of different materials determines the surgical efficacy and prognosis of patients to a certain extent. This article provides an overview of a variety of implant materials used for anterior column reconstruction after cervical spine tumor resection, introduces and analyzes their properties, advantages, disadvantages, derivatives, and applications in clinical practice, and looks forward to the future development of implant materials.

## Graphical abstract

This article provides an overview of a variety of implant materials used for reconstruction after cervical spine tumor resection, introduces and analyzes their properties, advantages, disadvantages, derivatives, and applications in clinical practice. They all play an essential role in maintaining the mechanical stability of cervical spine and improving patient outcomes after surgery and we cannot determine which one is best without a specific condition. The best implant material is still being explored.

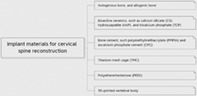

## Introduction

In clinical practice, primary spine tumors are rare, while most spine tumors are metastatic. Bone is the third most common site for tumor metastasis, next only to the lung and liver, and the spine is the most common site of bone metastases.^1^, ^2^, ^3^ Primary cervical spine tumors mainly include osteoid osteoma, osteoblastoma, chordoma, and plasmacytoma.^4^ The most common primary tumors that metastasize to the cervical spine are breast, lung, and prostate tumors.^5^ There are many differences in the pathological features of different types of cervical spine tumors. Approximately 40% of patients with cancer will develop spinal metastatic diseases, and 5%–10% will finally develop symptomatic epidural spinal cord compression.^6^ When the tumor compresses the spinal cord, it is possible to cause a series of adverse neurological symptoms,^7^ such as paralysis, pain, and even death in severe cases.^8^, ^9^ At this point, surgical intervention is usually required.^10^ In the past, the main surgical treatment for cervical spine tumors with spinal cord compression was decompressive laminectomy.^11^ In recent years, it has gradually evolved into a more direct anterior approach called anterior cervical corpectomy,^12^ involving removal of the vertebral body, reconstruction and stabilization. It offers a more direct approach for decompression of neural elements, tumor excision, and reconstruction of the weight‐bearing vertebral column for the majority of cervical spine tumors.^13^ Although surgical treatment may not cure the disease, it can relieve pain, restore stability, improve ambulatory function, and preserve neurologic function.^14^ Therefore, scientists are exploring in the field of implant materials for anterior column reconstruction.

The implant material must allow the surrounding bone to grow and adapt to its new adjacent structure. Ideally, it should have properties including biocompatibility, elastic modulus similar to that of bone, imaging and radiotherapy compatibility, optimal biomechanics, low artifacts on imaging, etc.,^15^, ^16^ but this is not the case in practice. For example, autogenous bone may cause many complications in the donor site; metal materials such as titanium mesh cage (TMC) may generate artifacts, which may hinder optimal tumor evaluation; polyetheretherketone (PEEK) is not conducive to osteoblast adhesion due to bioinertia. Therefore, more suitable implant materials are still being explored. Currently, implant materials frequently used for anterior column reconstruction include autogenous and allogenic bone, bone cement, TMC, expandable cervical cage (ECC), PEEK, or a combination of the above.

## Methods

Literature was identified by searching the PubMed and Google Scholar databases. The following MeSH (Medical Subject Heading) terms were included in our search strategy: “spinal neoplasms,” “surgery,” “bone cement,” “polymethyl methacrylate,” “titanium,” “polyetheretherketone,” “carbon fiber,” “porosity,” “elastic modulus,” “weight‐bearing,” “artifacts,” and “osseointegration.” The free text words included “cervical spine tumor,” “implants,” “bone graft,” “autogenous bone,” “allogeneic bone,” “bioactive ceramics,” “PMMA,” “titanium mesh cage,” “TMC,” “PEEK,” “3D‐printed,” “biocompatibility,” “bioactivity,” “porous,” “surface coating,” “subsidence,” “fatigue strength,” and “stress shielding.” The literature we reviewed included the following article types: review articles, systematic reviews, case reports, and original research studies. Among them, articles that were related to our topic and involved implant materials and/or cervical spine tumor were considered. Literature that involved implant materials but did not elaborate on the application to a specific field was excluded from consideration. Articles not relevant to our topic or in which the full text were not available were also excluded. Additionally, we reviewed the shortlisted literature from the search results to extract potentially suitable articles for this review. All publications were written in English. We also made a table for comparison of the advantages and disadvantages of different implant materials (Table 1).

**TABLE 1:** A summary of the advantages and disadvantages of different implant materials

Implant materials	Advantages	Disadvantages
Autogenous bone	Easy sampling	Donor site pain
	Good biocompatibility	Infection
	Complete histocompatibility	Long operative time and bleeding
	Without immune rejection	Subsidence
	High fusion rate	Pseudoarthrosis
		Insufficient supply
Allogenic bone	Sufficient supply	Poor bioactivity
	Shorter operative time	Immune rejection
	Less bleeding (compared to autogenous bone)	Transmission of infectious disease
	High fusion rate	Poor healing
	Relatively inexpensive	Aseptic loosening
Bioactive ceramics (CS, HAP, TCP)	Excellent bioactivity	Lack plasticity
	Good biodegradability	
	Good biocompatibility	
Bone cement (mainly PMMA)	Good plasticity	Toxicity
	Short setting time	Graft dislodgement
	Relatively inexpensive	Esophageal perforation
		Thermal damage to the spinal cord
		Compress surrounding tissues
TMC	Maintain the height of vertebral body	cage migration
	Immediate stability	Subsidence
	Good biocompatibility	High elastic modulus
	High fusion rate	Stress shielding
		Artifacts during imaging
PEEK	Moderate modulus of elasticity	Bioinert
	Optimal loading	Micromotion
	Without stress shielding	Relatively expensive
	Good chemical resistance	
	Radiolucent	
	Suitable mechanical property	
3D‐printed vertebral body	Personalized customization	Relatively expensive
	Low subsidence	
	Low pseudoarthrosis	
	Maintain intervertebral height	
	Maintain cervical physiological curvature	

Abbreviations: CS, calcium silicate; HAP, hydroxyapatite; PEEK, polyetheretherketone; PMMA, polymethylmethacrylate; TCP, tricalcium phosphate; TMC, titanium mesh cage.

## Autogenous Bone and Allogenic Bone

In orthopedics, a bone graft is usually used to provide an osteoconductive, osteoinductive, or osteogenic environment to promote bone repair and fusion. The clinical indications for the use of a bone graft mainly include malunion, nonunion, tumors that cause bone defects, avascular necrosis, etc.

### Autogenous Bone

Autogenous and allogenic bone grafts were among the first bone graft types used and are still frequently used today. Autologous bone graft means transplanting bone from one anatomical site of the patient to another site. It is considered an excellent bone graft material for cervical spine anterior column reconstruction due to its advantages of easy sampling, good biocompatibility, excellent osseointegration, etc. It was even the gold standard for anterior cervical spine surgery. Ilium bone is the most common site.

Autogenous bone is osteogenic, retains complete histocompatibility, provides structural support, and has no risk of disease transmission.^17^ Compared with allogenic bone, immune rejection will not occur as well. In fresh autogenous bone, surviving cells including osteocytes, osteoblasts, and mesenchymal cells can form new bone, increasing the probability of successful fusion, which is the most prominent advantage. Park *et al*.^18^ performed double‐segmental anterior cervical decompression and fusion with autogenous bone in 32 patients and recorded fusion rates, which were 28.1%, 68.8%, 93.8%, and 93.8% at 3, 6, 12, and 24 months respectively.

However, autogenous bone grafts may also cause donor site pain (the most common complication), infection, increased operative time and bleeding, and gait disturbance.^19^ Complications related to autogenous iliac bone graft include bone resorption, vertebral body subsidence, and pseudoarthrosis formation.^20^ Sayama *et al*.^21^ found that harvesting the iliac crest for grafting significantly resulted in postoperative pain and morbidity, affecting patients' life quality. They also claimed that using autogenous bone for anterior column reconstruction should be limited to patients with an expected survival of more than 6 months. Roberts *et al*.^22^ created a trephine harvest technique that provided ample autogenous bone without any complications or obvious pain at the harvest site during either short‐ or long‐term follow‐up, while their study was subject to historical controls for comparison. In contrast, the avoidance of donor site complications can be avoided by using allogenic bone.

### Allogenic Bone

Allogenic bone graft was first applied to clinical practice in 1881. It solves the major problem of insufficient autogenous bone supply in patients. Initially, the development of clinical application of allograft bone graft was limited due to poor bioactivity, immune rejection of the recipient, transmission of infectious diseases, poor healing, aseptic loosening, and insufficient donors.^23^ The above problems have been solved with the progress of allogenic bone processing and preservation technology, the development of bone bank construction, and the establishment of relevant management systems. According to different treatment methods, allogenic bone can be classified as fresh allogenic bone, deep‐frozen bone, fresh frozen allograft (FFA), and demineralized bone matrix (DBM). Fresh allogenic bone refers to the direct graft of the donor after removal without any treatment. Its disadvantages include easy to cause immune rejection of the recipient, and increased risk of transmission of infectious disease. Therefore, it has been eliminated in clinical surgery. Deep‐frozen bone refers to a bone graft processed from allogenic bone, trimmed into different shapes as needed, and then stored at low temperatures to retain the original mechanical strength. FFA is obtained by separating and thawing frozen bone to dehydrate it sufficiently and keep the water content in bone tissue below 5%, while the corresponding stress intensity is lost to some extent. DBM is obtained by using a series of chemical methods to decalcify and decrease allogenic bone to reduce immunogenicity while retaining a variety of osteogenic factors such as bone morphogenetic proteins to induce osteogenesis. It has a porous structure and is easier to combine with cytokines thus promoting bone regeneration.^24^ Although the structural strength decreases and the bearing capacity of bone is lost, it has unique clinical advantages in the repair and filling of bone defects.

Compared with autogenous bone, allogenic bone has advantages in terms of operative time and bleeding control, and donor site complications when using autogenous bone will not occur, just as we mentioned before. Allogenic bone is plentiful in supply and relatively inexpensive.^25^ Sun *et al*.^26^ reconstructed a patient's cervical spine with a mesh cage filled with allogenic bone graft to treat primary leiomyosarcoma of the cervical spine invading the vertebra. There were no recurrences 6 months after operation, and the reconstruction effect was good. Martin *et al*.^27^ used a freeze‐dried fibula allograft for reconstruction after anterior cervical discectomy and found that the allogenic fibula revealed a high fusion rate with minimal complications.

## Bioactive Ceramics

With the development of biomechanics and materials science, bone grafting methods become increasingly diverse. The implantation of artificial bone substitutes to repair bone defects and reconstruct spine has been a research hotspot. Artificial biomaterials that can replace human bone mainly consist of polymer synthetic materials, such as bioactive ceramics. Bioactive ceramics such as calcium silicate (CS), hydroxyapatite (HAP), and tricalcium phosphate (TCP) have been profusely studied for their ability to form direct bonds with living bone after implantation in bone defects.^28^ CS is the major ingredient of natural bone with excellent bioactivity and can bond to living bone and soft tissue.^29^ HAP can form new bone tissue binding to bone tissues through chemical bonds.^30^ TCP can enhance stem cell proliferation capacity and has good biodegradability, biocompatibility, and bioactivity.^31^ However, it is sintered at high temperatures in lumpy or granular form and lacks plasticity. During operation, the doctor cannot arbitrarily shape the material according to the bone defect of patients, and cannot completely fill the abnormal bone cavity. Therefore, these materials are usually used as surface coatings to improve the properties of PEEK, such as osseointegration and bioactivity.^32^ Frankenberger *et al*.^33^ found that the HA/SiO2‐based bioactive coating with interfacial composites on PEEK implants provided a lasting bone‐implant interface, and may be conducive to improving the property of bioinert surface of PEEK‐based implants. Addai *et al*.^34^ successfully prepared the reduced graphene oxide hydroxyapatite composite coating, significantly improving the hydrophilicity and bioactivity of PEEK.

## Bone Cement

As a cementing material, bone cement can be used in artificial joint replacement surgery and vertebral reconstruction surgery. It solves the problem of lack of plasticity in bioactive materials and can change from viscous state to solid state in approximately 15 min. Additionally, the short setting time enables patients to get out of bed earlier and reduce the time of bed rest. Bone cement can be divided into two categories: one is polymethylmethacrylate (PMMA) cement, which is a non‐degradable bioinert material, and the other is calcium phosphate cement (CPC), which is degradable. PMMA is used more widely in clinical practice.^35^ PMMA‐assisted reconstruction achieves immediate stabilization after radical tumor resection and is an advisable bone graft option for patients with limited life expectancy requiring further treatment. PMMA negates the need for postoperative external orthosis, avoids donor site complications, is unaffected by tumor invasion, and is inexpensive. Anchoring PMMA to the vertebral body securely can achieve better results.

However, PMMA has toxicity, together with the possibility of graft dislodgement, esophageal perforation, or even thermal damage to the spinal cord. Once it leaks, it is possible to compress the surrounding tissues. Allergic reaction to the components of bone cement may occur if patients have an allergic constitution. In severe cases, adverse effects such as shock may also occur. Long‐term problems include tumor regrowth that may necessitate repeat surgery. To solve thermal damage to the spinal cord, Xia *et al*.^36^ developed Paraffin/P(MMA‐MBA) PCM using the method of emulsion polymerization. They found that doping PMMA with microcapsules decreases its T_max_ and thus avoids the adverse effects of strong exothermicity during the PMMA setting process. Additionally, other techniques with PMMA have also been developed to prevent thermal injury to the underlying spinal cord. Cooper *et al*.^37^ first reported the use of PMMA‐filled silicone rubber tubing for thoracic and lumbar spine reconstruction. The technique placed key holes into the adjacent vertebral bodies and inserted a chest tube (impregnated with PMMA) into these holes. Shaaya *et al*.^38^ performed a C6‐C7 partial vertebrectomy for a patient with follicular thyroid carcinoma metastasized to the cervical spine using a posterior approach with posterior decompression and fusion from C2 to T2. The anterior column support was provided by a chest tube/PMMA construct, allowing the implant to be placed within the anterior column during the posterior approach without sacrificing the nerve roots. The patient tolerated it well, and no postoperative neurological deficit was noted. Later, Miller *et al*.^39^ used this PMMA reconstruction technique for patients with metastasized cervical spine tumors. A total of 29 patients underwent the coaxial double‐lumen PMMA technique followed by a subsequent anterior cervical plate, and the spinal cord of patients were protected well.

## Titanium Mesh Cage

TMC is one of the most common metal implants for cervical spine anterior column reconstruction. The cage constructs can restore the height of vertebral body and correct lordosis without obtaining autogenous bone from other sites. In anterior cervical corpectomy decompression and fusion surgery, using TMC combined with a local bone graft avoids bone donor site complications, and maintains immediate stability of the anterior column, together with biocompatibility and high fusion rate. TMC combined with autogenous bone graft has been generally accepted and used. Thalgott *et al*.^40^ also achieved a 100% fusion rate using TMC combined with a local bone graft in multilevel cervical corpectomy fusion. Additionally, Kang *et al*.^41^ proposed a novel method: a flanged TMC, which is also an effective graft for cervical spine anterior column reconstruction.

TMC has shown good efficacy in corpectomy, but placing the corpectomy defect *in situ* has proven difficult. Non‐expandable TMC may result in excessive distraction and weak compression. Excessive distraction may lead to segmental nerve stretch, injury, placing excessive pressure on the endplate, and increasing the risk of subsidence. Weak compression may lead to cage migration. An expandable TMC, which determines the height of the cage with proper compression but without excessive distraction of the adjacent vertebral body can solve these problems.^42^ Expandable cage directly applies and maintains the distraction force with a simple one‐step kyphosis‐correcting device without dangerous impaction (necessary with single‐height devices) on the spinal cord, and can also span multiple segments. Other types of expandable cages such as Synex ECC, have a self‐locking ratchet expansion mechanism for easy insertion into the vertebral body defect, and provide different heights and endplate sizes for the cervical, thoracic, or lumbar spine. And it was used for a case of cervical spine tumor by Auguste *et al*.^43^ who found that ECC was suitable for cervical spine anterior column reconstruction and correction of sagittal malalignment.

**Fig. 1:**
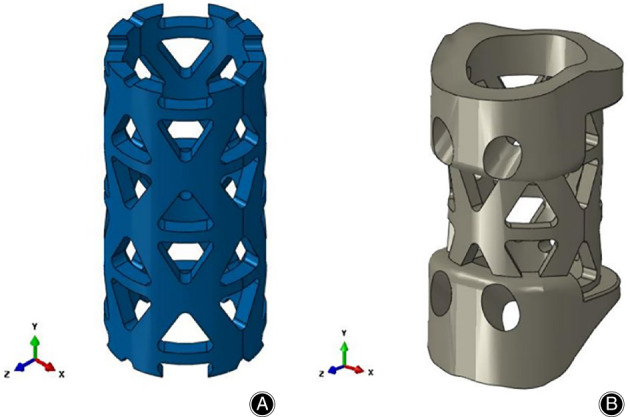
Two titanium mesh cage models. (A) Traditional titanium mesh cage (TTMC); (B) novel titanium mesh cage (NTMC) (Solidworks 2016, SOLIDWORKS, Co, USA)^48^ (Taken from Wang *et al*.^48^)

Other disadvantages of TMC include the potential of cage migration, fracture, and subsidence. Severe subsidence may cause symptoms recurring, neurological function deterioration, internal fixation failure, and cervical kyphosis.^44^ The incidence of postoperative TMC subsidence was reported to be over 28.3%.^20^ TMC is embedded in the endplate with a dentate edge once implantation, while the adhesiveness is poor. The contact area between TMC and the vertebral endplate is point‐to‐face and limited, resulting in uneven stress distribution and relatively concentrated stress, thus leading to postoperative TMC subsidence. However, the definition of TMC subsidence is controversial. Van Jonbergen *et al*.^45^ suggested that due to measurement errors, a decrease in postoperative intervertebral height of over 3 mm is defined as TMC subsidence. Chen *et al*.^46^ classified TMC subsidence as mild subsidence (1–3 mm) and severe subsidence (>3 mm). In an 8‐year follow‐up study, the TMC subsidence of >3 mm was approximately 40.4%.^47^ Wang *et al*.^48^ proposed a novel type of anatomical TMC (Fig. 1) with a curved structure of the upper and lower edges completely conforming to the upper and lower end of the cervical disc and achieving face‐to‐face contact of the cervical spine, and it was reported to greatly improve the anti‐subsidence performance, 51.3% higher than that of traditional TMC.

Apart from subsidence, TMC, as a metal material, also causes artifacts and stress shielding. Artifacts affect doctors' judgment on tumor recurrence and efficacy after tumor resection. The higher elastic modulus of TMC than that of cortical bone is the main reason for stress shielding. According to Wolf's law,^49^ high elastic materials result in a decrease in bone density in the peri‐implant area. Tytgat *et al*.^50^ reported that bone remodeling is highly sensitive to dynamic loading. Due to stress shielding, developing materials with lower elastic modulus and mechanical resistance in short‐ and long‐term becomes necessary.

## Peek

As a high‐performance thermoplastic, PEEK solved the problem of high elastic modulus of metal implants and was commercially supplied from April 1998.^51^ The first application of PEEK as spinal implant was as an intervertebral cage, which overcame two problems of traditional metal intervertebral cages: high elastic modulus and stress shielding. The elastic modulus of PEEK (approximately 3.6 GPa) is smaller than that of cortical bone (17–21 GPa), and carbon fiber can be added into PEEK to make its elastic modulus closer to that of cortical bone.^52^ This property allows optimal loading of the bone and prevents stress shielding. Moreover, with good chemical resistance,^53^ PEEK can resist the body's natural oxidative environment, thus minimizing the chance of local tissue reactions. Moreover, PEEK is radiolucent and resistant to radiation damage,^54^ which is convenient for facilitating radiographic assessment of implants fusion results,^55^ and suitable for postoperative radiotherapy.^44^

However, PEEK is a kind of bioinert material due to its hydrophobic chemistry of the surface, which is not conducive to osteoblast adhesion. Creating an osteogenic environment by modifying the surface structure can promote bone formation and implant stability without adding exogenous growth factors. Therefore, HAP whiskers, HAP microparticles, and other ceramic particulates have been incorporated into PEEK to enhance the bioactivity such as osteocyte adhesion and mechanical stiffness.^56^ We also mention this when we introduce bioactive ceramics. On the other hand, PEEK cannot fuse well with the surrounding bone and might form a fibrous junction interface,^57^ so subsequent micromotion may occur, causing reconstruction failure. To improve the properties of PEEK, a variety of PEEK composites such as Nano‐TiO2/PEEK,^58^, ^59^ and Nano‐HAp/PEEK,^60^, ^61^ have also been developed to promote cell adhesion, proliferation and osteogenesis. Moreover, PEEK/Carbon improves the mechanical property and crystallization rate of PEEK.^62^, ^63^, ^64^ Boriani *et al*.^65^ used composite PEEK/carbon fiber rods to treat cervical spine tumor and reported that hybrid implants were an effective solution for cervical and cervicothoracic segment reconstruction. And the implants did not produce artifacts in postoperative images, alleviating the execution of postoperative radiotherapy. Pipola *et al*.^66^ reported a case of sclerosing epithelioid fibrosarcoma at C7 involving the right posterior cervical region from C5 to right lung apex. To ensure the stability of the cervical spine and the effect of particle treatment, they proposed a new technology of carbon fiber reinforced peek preformed rods with a sublaminar belt to fix the cervicothoracic junction. During a 2‐year follow‐up, the implant was stable, and no local recurrence or mechanical failure were noted. However, currently, the properties of PEEK‐based implant materials affecting bioactivity and postoperative stability have not been completely optimized.

## 3D‐Printed Vertebral Body

3D‐printed technology is an innovative technology and has undergone substantial development in the field of spine surgery.^67^ Its unique production process allows for customization and rapid prototyping of complex geometries, which are impossible for traditional manufacturing to achieve. Engineers can reconstruct the damaged bone of patients through computer modeling using CT, MRI, or other medical images, and create prostheses with mechanical and biological properties that better match to the bones, which may produce lower subsidence and pseudoarthrosis rate.^68^ 3D printed vertebral body customized according to different patients is a breakthrough in implant materials for anterior column reconstruction. Due to the unique process of 3D‐printed technology, the surface of 3D‐printed vertebral body is relatively rough, creating a more ideal condition for early cell adhesion.

Compared with TMC, 3D‐printed vertebral body has better performance in maintaining intervertebral height and cervical physiological curvature.^69^ Yang *et al*.^70^ used a personalized 3D‐printed vertebral body produced from titanium alloy for cervicothoracic reconstruction of a six‐segment recurrent chordoma and claimed that it had a better load‐bearing capacity against subsidence or dislocation, which effectively reduced the rate of revision in the long‐ term. Xu *et al*.^71^ employed a personalized 3D‐printed vertebral body (Fig, 2)^72^ fabricated through computer model using titanium alloy powder to reconstruct the upper cervical spine of a teenager with Ewing sarcoma. The microstructure was optimized through the designs of optimized pore diameter and pore density of pore metal structure to gain better biomechanical stability and enhance bone healing. This was the first successful case of the clinical application of 3D‐printed titanium alloy orthopedic implant.

**Fig. 2:**
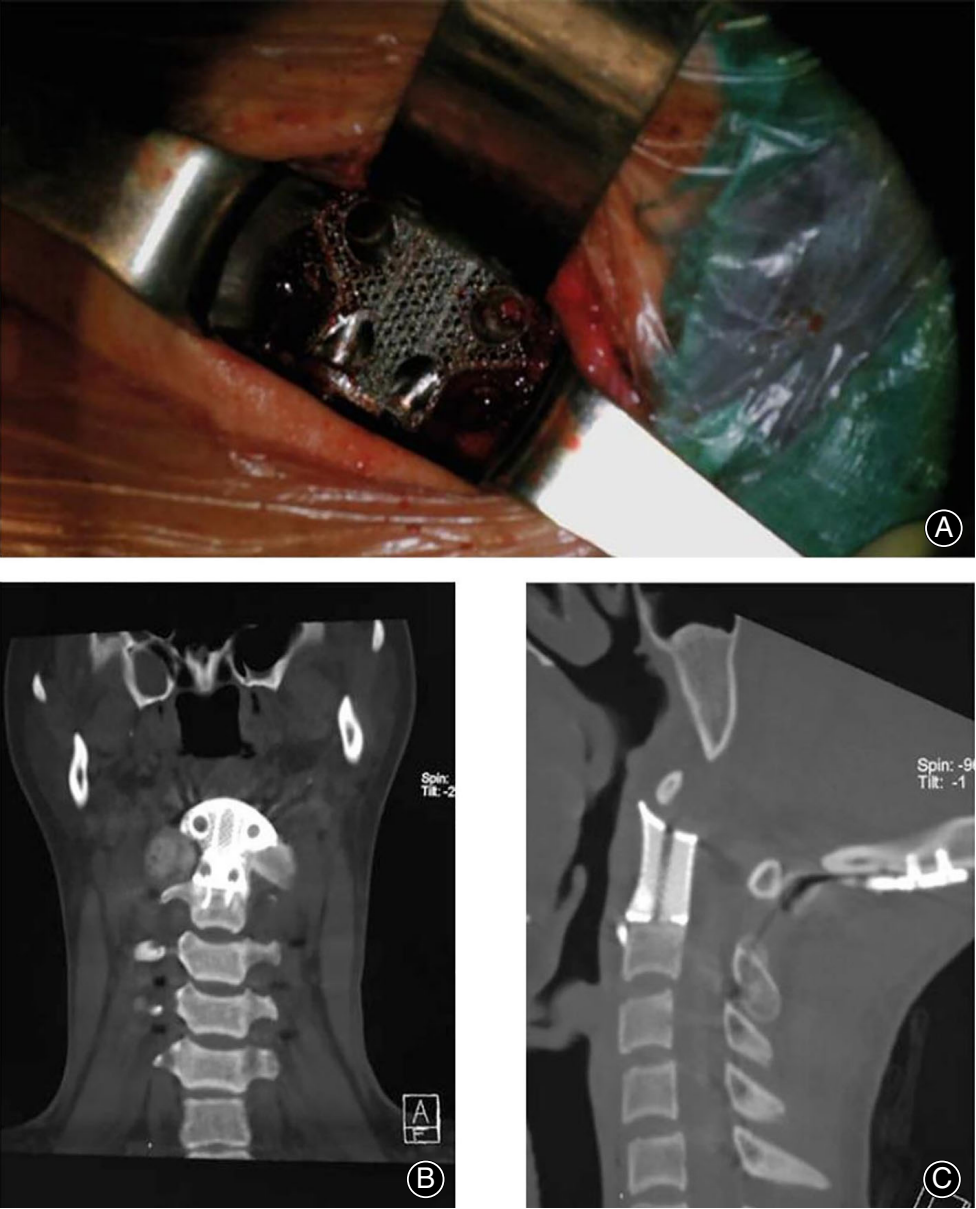
(A) Intraoperative view of the 3D‐printed artificial vertebral body in place during the anterior procedure. (B) Coronal CT reconstruction of the 3D‐printed artificial vertebral body at the 3‐month follow‐up visit. (C) Sagittal CT reconstruction of the 3D‐printed artificial vertebral body at the 3‐month follow‐up visit.^72^ (Taken from Cai *et al*.^72^)

Additionally, the mechanical rigidity and elasticity of 3D‐printed PEEK are close to those of human bone,^73^ and it is transparent to X‐rays. Given PEEK's bioinertia and thus limited interaction with bone, porous PEEK perfectly solves this problem. Controlling porosity can significantly improve the material osteoconduction. Godlewski *et al*.^74^ thought that producing 3‐dimensional porous‐surfaced implants opens up considerable prospects for this technique in the production of modern interbody implants. 3D‐printed porous PEEK is related to increased preosteoblast activity compared with 3D‐printed solid controls.^75^, ^76^ Vijayavenkataraman *et al*.^77^ found that in biological testing, the size of the pores can affect the response of cells, while for bone ingrowth, a size gradient is recommended. Spece *et al*.^75^ successfully fabricated porous PEEK scaffolds with pore size and porosity similar to trabecular bone using an extrusion‐based and commercialized 3D printing technique, but they did not conduct animal or human experiments to further verify the bone ingrowth ability of 3D‐printed porous PEEK. And other literature related to 3D‐printed porous PEEK has no similar reports yet. In the future, if the bone ingrowth of 3D‐printed porous PEEK is verified efficiently through animal or human experiments, it will make a major breakthrough in the field of implant materials for cervical spine anterior column reconstruction.

### Conclusion

Surgery combined with postoperative radiotherapy has become the main treatment modality for cervical spine tumors. In recent years, implant materials for cervical spine anterior column reconstruction after tumor resection have been extensively studied and used with varying degrees of success in orthopedic surgery. Implant materials have undergone a constant evolution and developed from original autogenous bone to bioactive materials, metal materials, organic polymer materials, and now personalized 3D‐printed vertebral body. We cannot easily determine which one is the best without a specific condition. They all play an essential role in maintaining the mechanical stability of the cervical spine and improving patient outcomes after surgery. Currently, the most popular implant materials are TMC and PEEK. The best implant material is still being explored. Looking ahead, continued in‐depth research on implant materials will be beneficial to the further development of the field of cervical spine anterior column reconstruction. Spine surgeons should keep an eye on the current literature on implant materials.

## Author Contributions

All authors contributed to the review conception and design. Xiaoguang Liu and Zhongjun Liu decided the content and framework of the article. Xiao Liu, Yan Li, and Panpan Hu were the guarantors of the overall content. Jiasheng Chen, Shuheng Zhai, and Hua Zhou wrote the first draft of the manuscript. Zihe Li and Feng Wei revised the manuscript.

## Conflict of Interest Statement

The authors declare that they have no conflicts of interest.
